# Supplementary material for: Production and Characterization of a Novel Exopolysaccharide from Ramlibacter tataouinensis
Source: Molecules. 2022 Oct 24;27(21):7172. doi: 10.3390/molecules27217172 (PMC9658432; doi:10.3390/molecules27217172)
Supplement: Supplementary file 1 [file molecules-27-07172-s001.zip › molecules-1935737-supplementary.pdf]

Production and characterization of a novel exopolysaccharide from *Ramlibacter tataouinensis*

Desislava Jivkova[1,2#], Ganesan Sathiyanarayanan[1,2#], Mourad Harir[3,4], Norbert Hertkorn[3], Philippe Schmitt-Kopplin[3,4], Ghislain Sanhaji[5], Sylvain Fochesato[1,2], Catherine Berthomieu[6], Alain Heyraud[7], Wafa Achouak[1,2], Catherine Santaella[1,2*], Thierry Heulin[1,2*]

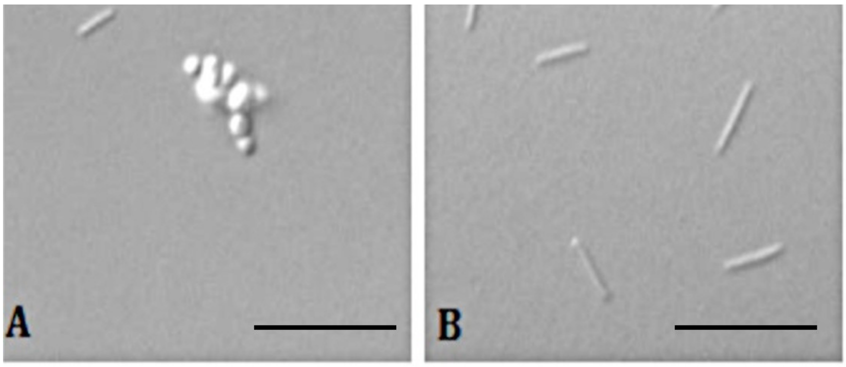

Figure S1. Cellular morphology of *R. tataouinensis*. (A) Cyst form of *R. tataouinensis* and (B) Rod-shaped *R. tataouinensis* when growing in TSB/10 medium at 30 °C for 3 days in the dark. These observations were carried out by bright-field optical microscopy. Scale bar represents 20 µm.

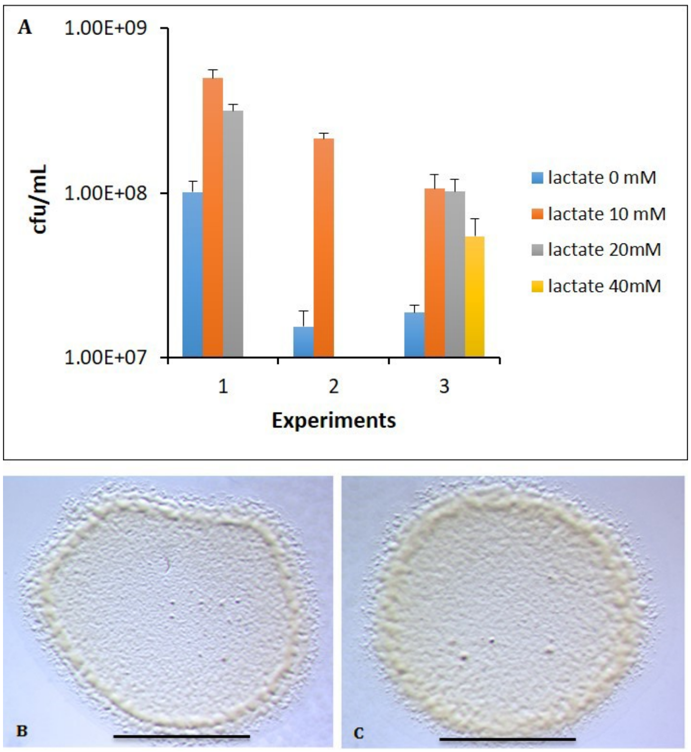

Figure S2. Estimation of the growth of *R. tataouinensis* in presence of lactate: (A) in TSB 1/10 medium with 10, 20- or 40-mM lactate (incubation with stirring 150 rpm, at 30 °C for 3 days) by counting cfu/mL (inoculum: 1%, 10^5-10^6 cfu/mL). Error bars are the standard deviations of the mean. and *R. tataouinensis* colonies on TSA 1/10 with (C) or without (B) 10 mM lactate after four days on incubation under dark at 30 ° C. Scale bar: 4 mm

A

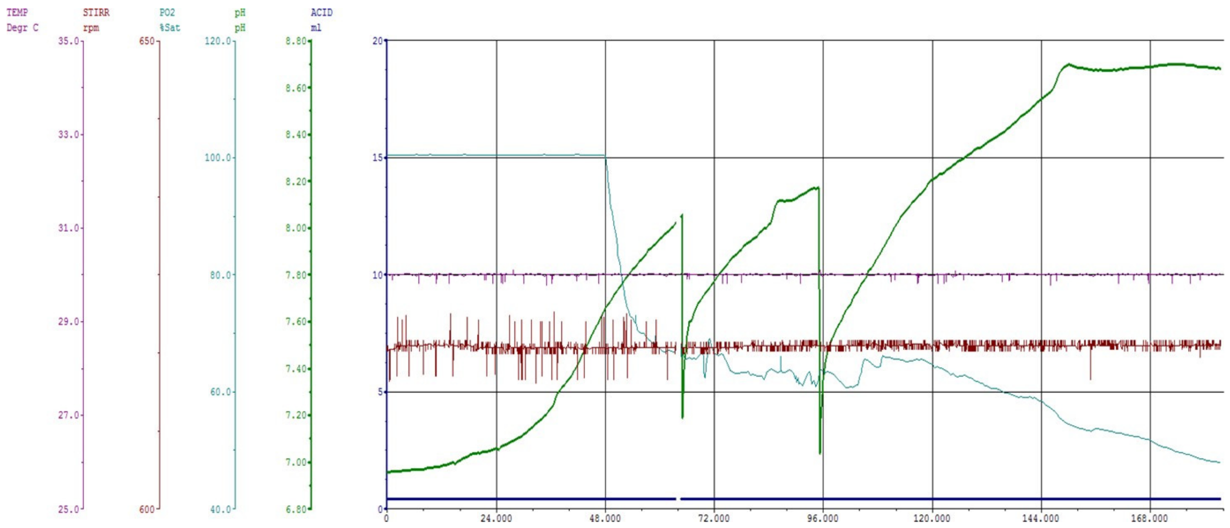

B

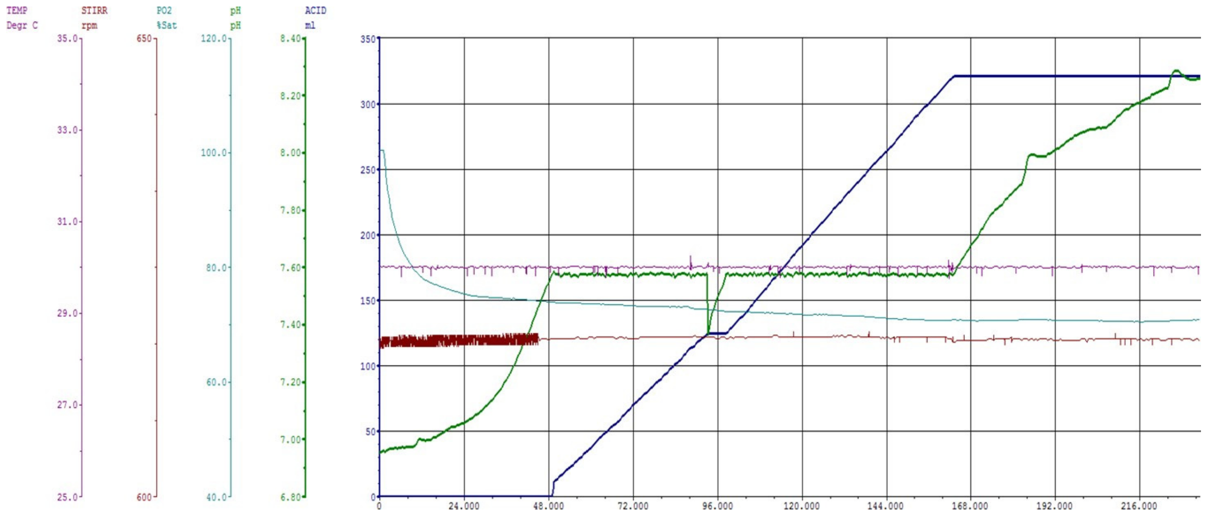

Figure S3. Optimization of cysts formation by fed-batch fermentation in bioreactor: The graph shows

the pH, pO_2 (DO), temperature and agitation curves. (A) First experiment of development of *R. tataouinensis* in TSB 1/10 culture medium supplemented with 10 mM lactate in a bioreactor. Two injections of sterile and concentrated lactic acid (80%, final concentration 2.2 mM) were made at 65 h

and 95 h of fermentation. (B) The pH was regulated by continuous lactic acid (LA) injection 10%. A TSB intake (1 g/L). The bioreactor was mainlined with sterile air supply of 0.2 vvm, agitation at 1.98 m/s and culture temperature of 30 °C.

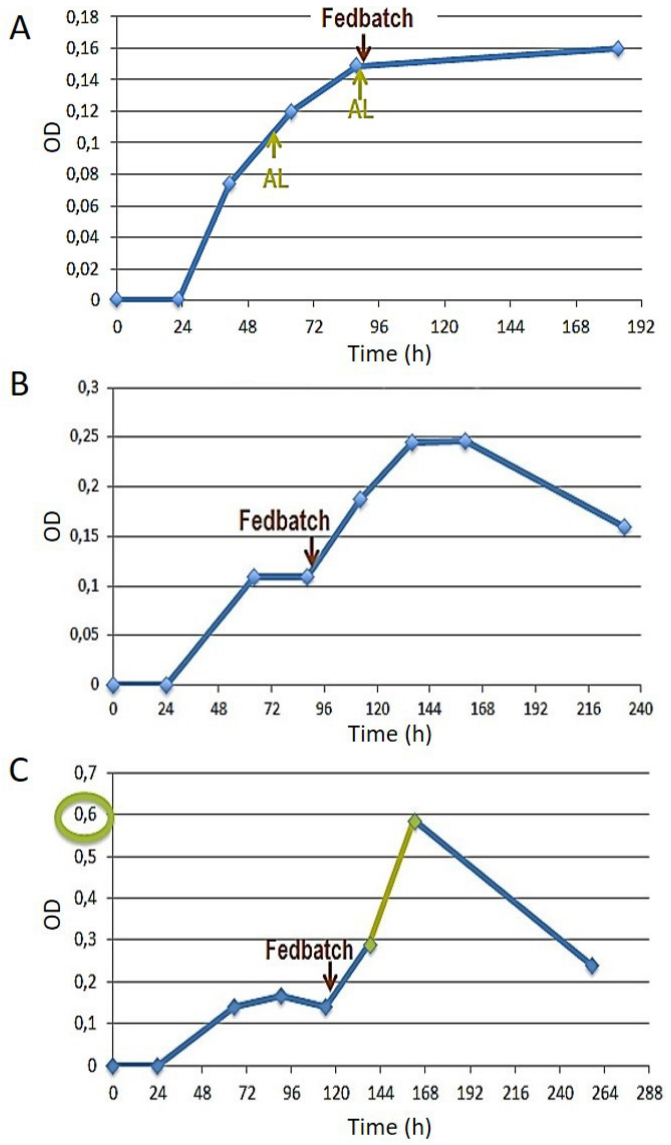

Figure S4. Growth (OD at 600 nm) of *R. tataouinensis* in TSB 1/10 medium supplemented with 10 mM lactate under fed-batch fermentation: (A) Experiment without pH regulation and addition of lactic acid (AL) and carbon substrate (TSB, 1 g/L) are indicated by arrows. (B) Experiment with pH regulation, in which lactic acid (10%) was introduced to regulate the pH (pH 7.6 set point) along with TSB (1 g/L) on the fourth day. (C) Experiment with optimized medium with continuous pH regulation and tryptone 3 g/L and yeast extract 1 g/L were added on the 5th day of fermentation. The overall processes were maintained with sterile air supply (0.2 vvm), agitation at 1.98 m/s, and temperature at 30 °C.

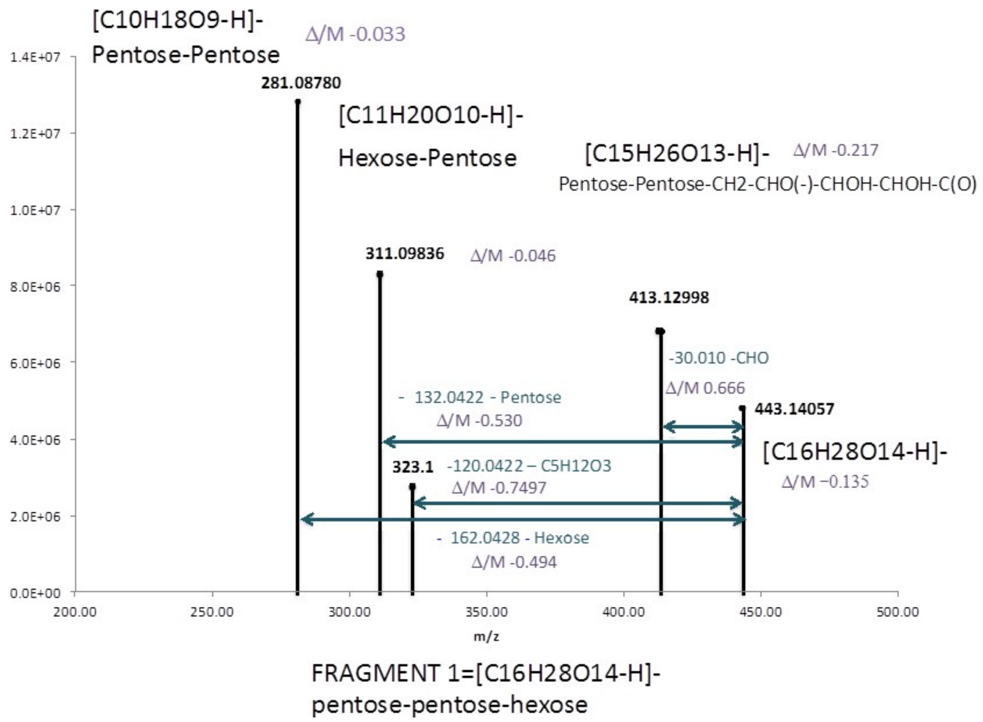

Figure S5. Example of interpretation of m/z and mass loss between m/z for a 'pentose-pentose-hexose' oligosaccharide (mass 443.14057). The tolerance between theoretical and experimental mass for both m/z and neutral mass loss is ± 0.7 ppm.

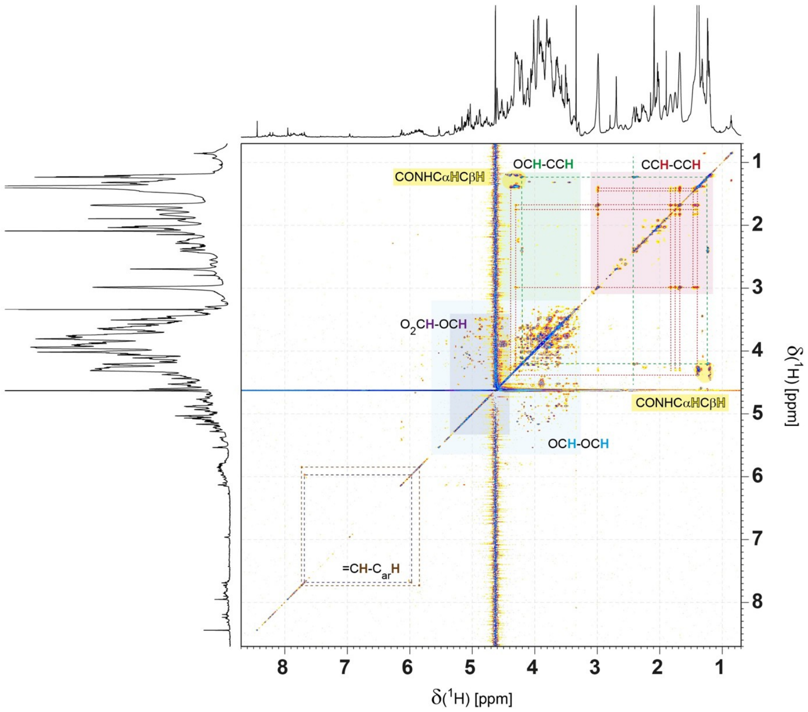

Figure S6. ^{1}H, ^{1}H TOCSY NMR spectrum (800 MHz, D_2O, 310 K, mixing time = 70 ms) of *R. tataouinensis* EPS; (**A**) CC**H**-CC**H** cross peaks within aliphatic section, cf. Fig. S10; (**B**) OC**H**-CC**H** cross peaks, connecting aliphatic and oxygenated units; (**C**) OC**H**-OC**H** cross peaks, representing largely carbohydrates; (**D**) O_2C**H**-OC**H** cross peaks within carbohydrates (blue shaded section, cf. Fig. S8; purple shaded region, cf. Fig. S9A).

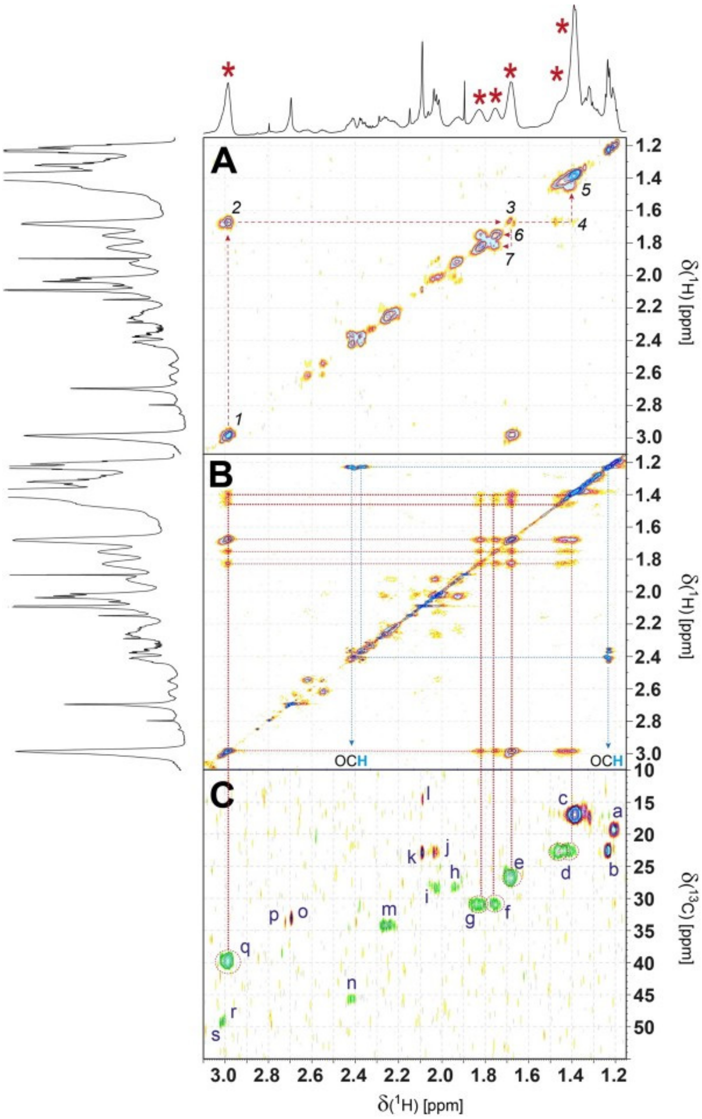

Figure S7. **(A)** ^{1}H, ^{1}H COSY NMR spectrum (800 MHz, D_2O, 310 K, mixing time = 70 ms), **(B)** ^{1}H, ^{1}H TOCSY NMR spectrum, and (**C**) CH_2-selective ^{1}H, ^{13}C DEPT HSQC NMR spectrum of *R. tataouinensis* EPS: aliphatic section; green: CH_2, red: CH_3 and CH units. Dotted lines denote a major contiguous, and probably a nitrogen-containing aliphatic unit. For annotation $\delta_{H/C}$ of HSQC cross peaks, see Table S7.

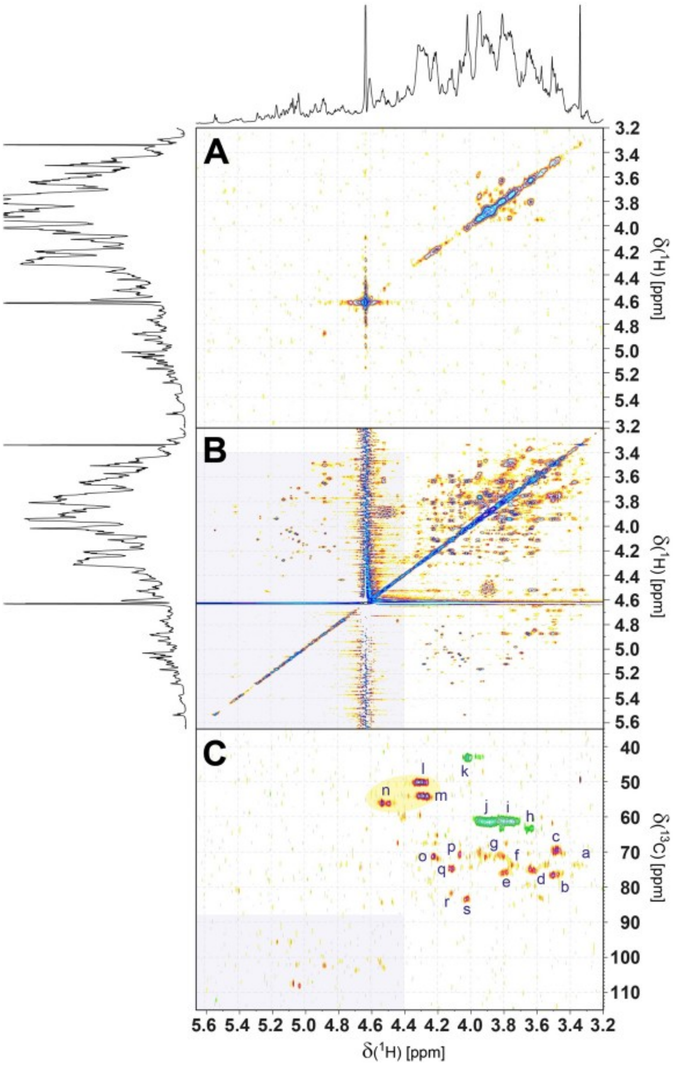

Figure S8. **(A)** ^{1}H, ^{1}H COSY NMR spectrum (800 MHz, D_2O, 310 K, mixing time = 70 ms), **(B)** ^{1}H, ^{1}H TOCSY NMR spectrum, and (**C**) CH_2-selective ^{1}H, ^{13}C DEPT HSQC NMR spectrum of *R. tataouinensis* EPS: section of oxygenated aliphatic units, *i.e.* carbohydrates; green: CH_2, red: CH_3 and CH units. More than 35 correlation peaks from anomeric protons confirm the presence of complex material. For annotation $8_{H/C}$ of HSQC cross peaks, see Table S6; yellow shaded section probably indicates CONH**<u>CaH</u>** cross peaks within peptide linkages.

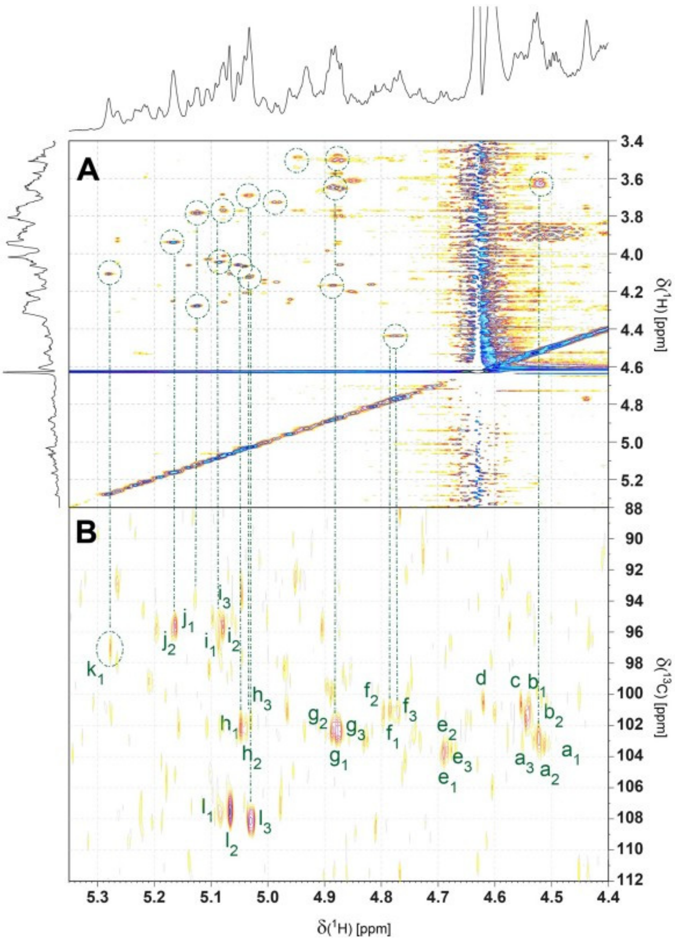

Figure S9. **(A)** ^{1}H, ^{1}H TOCSY NMR spectrum, and (**B**) CH_2-selective ^{1}H, ^{13}C DEPT HSQC NMR spectrum of *R. tataouinensis* EPS: section of anomeric units O_2**<u>CH</u>** within carbohydrates. The major cross peaks, and their $\delta_{H/C}$ values are depicted in Table S7.

Table S1. Acquisition parameters of NMR spectra, shown according to figures. NS: number of scans (for 2D NMR: F2); AQ: acquisition time [ms]; D1: relaxation delay [ms]; NE: number of F1 increments in 2D NMR spectra; WDW1, WDW2: apodization functions in F1/ F2 (EM/GM: line broadening factor [Hz]; QS: shifted square sine bell; SI: sine bell); PR1, PR2: coefficients used for windowing functions WDW1, WDW2, EM/GM are given in [Hz], SI/QS derived functions indicate shift by π/n.

spectrum	Figure (expno)	NS	AQ [ms]	D1 [ms]	NE	WDW1	WDW2	PR1	PR2
^{1}H NMR	1	4096	5000	5000	-	EM	-	1	-
^{1}H, ^{1}H COSY	S7, S8	32	1078	422	512	EM	QS	3	2.5
^{1}H, ^{1}H TOCSY	S6, S7, S8, S9	12	1078	1922	2048	EM	QS	2.5	4
^{1}H, ^{13}C DEPT HSQC	S6, S7, S8, S9	256	250	1250	215	EM	QS	2	2.5

Table S2. Ion characteristic of monosaccharides. For ease of reading, masses have been rounded to the unit.

Motif or substituent	Molar Mass	[M-H]-	[M -H_2O]	[M-H_2O-H]-
Hexose	180	179	162	161
Uronic Acid (UA)	194	193	176	175
Pentose	150	149	132	131
Deoxyhexose	164	163	146	145
Acetate	60	59	42	41

Table S3. Loss of mass corresponding to fragmentations of monosaccharides

Fragmentation	Mass losses (uma)	Raw formula
0.1A	30	CHO
0.2A	60	$C_2H_4O_2$
2.4A	120	$C_4H_6O_4$
0.4A	90	$C_3O_3H_6$

Table S4. Use of an m/z distance matrix to search for characteristic mass losses of mono- and oligosaccharides. For ease of reading, masses were rounded to the unit. In accordance with the nitrogen rule, the mass losses sought are even. Odd mass losses have been replaced by 0's in the matrix. The yellow boxes symbolize the diagonal of the matrix. Each mass loss is illustrated by a different formatting. For example, -162 loss of a hexose, -132 loss of a pentose, -42 loss of an acetate, -18 loss of H_2O.

m/z	801	785	783	783	719	571	443	439	427	425	423	413	407	405	395	377	359	339	337	323	311	309	307	293	291	291	281	275	263	175	161
**801**	0	0	0	18	0	0	0	362	0	0	378	0	394	396	406	424	442	0	464	478	490	492	494	508	510	510	520	526	538	626	640
**785**	0	0	0	2	0	0	0	346	0	0	362	0	378	380	390	408	426	0	448	462	474	476	478	492	494	494	504	510	522	610	624
**783**	0	0	0	0	0	212	0	344	0	358	360	370	376	378	388	406	424	444	446	460	472	474	476	490	492	492	502	508	520	608	622
**783**	+18	+2	0	0	0	0	0	0	0	0	0	0	0	0	0	0	0	0	0	0	0	0	476	0	0	0	0	0	0	608	622
**719**	0	0	0	0	0	148	276	280	292	294	296	306	312	314	324	342	360	380	382	396	408	410	412	426	428	428	438	444	456	544	558
**571**	0	0	+212	0	+148	0	0	132	0	146	148	158	164	166	176	194	212	232	234	248	260	262	264	278	280	280	290	296	308	396	410
**443**	0	0	0	0	+276	0	0	4	0	18	20	30	36	38	48	66	84	104	106	120	132	134	136	150	152	152	162	168	180	268	282
**439**	+362	+346	+344	0	+280	+132	+4	**0**	0	0	0	0	0	34	0	62	80	0	102	116	128	0	132	146	148	148	158	164	176	264	278
**427**	0	0	0	0	+292	0	0	0	0	2	4	14	20	22	32	50	68	88	90	104	116	118	120	134	136	136	146	152	164	252	266
**425**	0	0	+358	0	+294	+146	+18	0	+2	0	2	0	18	20	30	48	66	86	88	102	114	116	118	132	134	134	144	150	162	250	264
**423**	+378	+362	+360	0	+296	+148	+20	0	+4	+2	0	0	0	18	0	46	64	0	86	100	112	0	116	130	132	132	142	148	160	248	262
**413**	0	0	+370	0	+306	+158	+30	0	+14	0	0	0	6	8	18	36	54	74	76	90	102	104	106	120	122	122	132	138	150	238	252
**407**	+394	+378	+376	0	+312	+164	+36	0	+20	+18	0	+6	0	2	0	30	48	0	70	84	96	98	100	114	116	116	126	132	144	232	246
**405**	+396	+380	+378	0	+314	+166	+38	+34	+22	+20	+18	+8	+2	0	0	0	46	0	68	82	94	0	98	112	0	114	124	130	142	230	244
**395**	+406	+390	+388	0	+324	+176	+48	0	+32	+30	0	+18	0	0	0	18	36	0	58	72	84	86	88	102	104	104	114	120	132	220	234
**377**	+424	+408	+406	0	+342	+194	+66	+62	+50	+48	+46	+36	+30	0	+18	0	18	0	40	54	66	0	70	84	86	86	96	102	114	202	216
**359**	+442	+426	+424	0	+360	+212	+84	+80	+68	+66	+64	+54	+48	+46	+36	+18	0	0	22	36	48	0	52	66	0	68	78	84	96	184	198
**339**	0	0	+444	0	+380	+232	+104	0	+88	+86	0	+74	0	0	0	0	0	0	2	16	28	30	32	46	48	48	58	64	76	164	178
**337**	+464	+448	+446	0	+382	+234	+106	+102	+90	+88	+86	+76	+70	+68	+58	+40	+22	+2	0	0	0	0	30	0	0	46	0	62	74	162	176
**323**	+478	+462	+460	0	+396	+248	+120	+116	+104	+102	+100	+90	+84	+82	+72	+54	+36	+16	0	0	12	0	16	30	0	32	42	48	60	148	162
**311**	+490	+474	+472	0	+408	+260	+132	+128	+116	+114	+112	+102	+96	+94	+84	+66	+48	+28	0	+12	0	0	4	18	0	20	30	36	48	136	150
**309**	+492	+476	+474	0	+410	+262	+134	0	+118	+116	0	+104	+98	0	+86	0	0	+30	0	0	0	0	2	16	18	18	28	34	46	134	148
**307**	+494	+478	+476	+476	+412	+264	+136	+132	+120	+118	+116	+106	+100	+98	+88	+70	+52	+32	+30	+16	+4	+2	0	0	0	0	0	0	0	132	146
**293**	+508	+492	+490	0	+426	+278	+150	+146	+134	+132	+130	+120	+114	+112	+102	+84	+66	+46	0	+30	+18	+16	0	0	0	2	0	18	30	118	132
**291**	+510	+494	+492	0	+428	+280	+152	+148	+136	+134	+132	+122	+116	0	+104	+86	0	+48	0	0	0	+18	0	0	0	0	10	16	28	116	130
**291**	+510	+494	+492	0	+428	+280	+152	+148	+136	+134	+132	+122	+116	+114	+104	+86	+68	+48	+46	+32	+20	+18	0	+2	0	0	0	0	0	116	130
**281**	+520	+504	+502	0	+438	+290	+162	+158	+146	+144	+142	+132	+126	+124	+114	+96	+78	+58	0	+42	+30	+28	0	0	+10	0	0	6	18	106	120
**275**	+526	+510	+508	0	+444	+296	+168	+164	+152	+150	+148	+138	+132	+130	+120	+102	+84	+64	+62	+48	+36	+34	0	+18	+16	0	+6	0	0	100	114
**263**	+538	+522	+520	0	+456	+308	+180	+176	+164	+162	+160	+150	+144	+142	+132	+114	+96	+76	+74	+60	+48	+46	0	+30	+28	(	+18	0	0	88	102
**175**	+626	+610	+608	+608	+544	+396	+268	+264	+252	+250	+248	+238	+232	+230	+220	+202	+184	+164	+162	+148	+136	+134	+132	+118	+116	+116	+106	+100	+88	0	0
**161**	+640	+624	+622	+622	+558	+410	+282	+278	+266	+264	+262	+252	+246	+244	+234	+216	+198	+178	+176	+162	+150	+148	+146	+132	+130	+130	+120	+114	+102	0	0

Table S5. Attribution of potential structures corresponding to m/z and loss of mass between signals. UA symbolizes an uronic acid.

**439,10922**	[UA-Pentose-Pentose -H2O-H]-	C16H26O15-H2O-H]-	439,10933	439,10922	-0,25051
	[UA-Hexose-Hexose-C2H4O2-H2O-H]	C18H30O17-C2H4O2-H2O-H–	439,10933	439,10922	-0,25051
**427,16099**				427,16099	
	[Hexose-1-->6-(3,4-DiOAc)-Hexose-H] perte de masse de -102 (60+42), -132 (90+42), -144 (102+42), pertes -				
**425,12997**	18, -30, -60 -90 caractéristiques enchainement 1,6	[C16H26O13-H]-	425,13006	425,12997	-0,21170
	[Pentose-pentose-hexose-H2O-H]-	[C16H28O14-H2O-H]-	425,13006	425,12997	-0,21170
	[UA-6Deoxyhexose-6-deoxyhexose -C2H4O2-H]-	[C18H30O15-C2H4O2-H]-	425,13006	425,12997	-0,21170
	[Hexose-Hexose-Hexose -C2H4O2-H2O-1]-	[C18H32O16-C2H4O2-H2O-1]-	425,13006	425,12997	-0,21170
**423,11429**	Succinyl-Hexose-Hexose-H2O-H]-	[C16H26O14-H2O-H]-	423,11441	423,11429	-0,28361
	Succinyl-Hexose-Pentose -C2H4O2-H]-	[C16H30O16-C2H4O2-H]-	423,11441	423,11429	-0,28361
**413,12998**	[Pentose-Pentose-Pentose-H); fragments -18, -90 -102 -132	[C15H26O13-H]-	413,13006	413,12998	-0,19364
	[Pentose-Pentose-Hexose-C2H4O2-H); m/z 425-18-H; m/z 443 fragmentation de l'hexose-30 (-CH2O)	[C17H30O15-C2H4O2-H]-	414,13006	413,12998	-0,19364
**407,11943**	[Hexose-Hexose-Di-OAc -H2O-H]-	[C16H26O13-H2O-H]-	407,11950	407,11943	-0,17194
	[Hexose-1-->6-(3,4-DiOAc)-Hexose-H2O-H]				
**405,10376**	m/z 423 -18				
**395,11947**	[Tri-pentose-H2O-H]- ; m/z 425 0,2A ; m/z 443 fragmentation de l'hexose-60 (C2H4O2)	[C15H26O13-H2O-H]–	395,11950	395,11947	-0,07593
	[Pentose-Hexose-Hexose-C2H4O2-H2O-H]-	[C17H28O14-C2H4O2-H]-	395,11950	395,11947	-0,07593
	[Succinyl-Hexose-Hexose-OMe-H]-	[C17H28O14-C2H4O2-H]-	395,11950	395,11947	-0,07593
	[OMe-6-Deoxyhexose-Hexose-H]-	[C17H28O14-C2H4O2-H]-	395,11950	395,11947	-0,07593
**377,10891**	[Succinyl-Hexose-Hexose-OMe -C2H4O2-H]-] ou [Succinyl-Hexose-OAc-pentose -C2H4O2-H]-	[C17H28O14-C2H4O2-H2O-H]-	377,10891	377,10891	0,00000
**359,09840**					
**339,12966**	[OMethyl -6-deoxyhexose-Hexose -H]-	C13H24O10-H]-	339,12967	339,12966	-0,02949
**337,07757**	m/z 439 -102 (60+42, C4H6O3) 0,2A avec acétate en position 2 de l'hexose				
	[UA-Hexose -H2O-H]-	[C12H20O12-H2O-H]-	337,07763	337,07757	-0,17800
**323,0984**	[Hexose-Hexose-H2O-H]-	C12H22O11-H2O-H]-	323,09837	323,09838	0,03095
	[OAc-Hexose-Hexose-C2H4O2-H]-	[C14H24O12-C2H4O2-H]-	323,09837	323,09838	0,03095
	m/z 443 fragmentation de l'hexose -120 (C5H12O3) et m/z 413-90 (C3H6O3)		323,09837	323,09838	0,03095
	[Hexose-Hexose-OAc-OAc-C4H6O3-H] *0,2A*	[C16H26O13-C4H6O3-H]-	323,09837	323,09838	0,03095
**321,11907**	[OMethyl-6-deoxyhexose-Hexose -H2O-H]-	C13H24O10-H2O-H]-	321,11911	321,11907	-0,12456
**311,09836**	[Pentose-Hexose -H]- m/z 443 -pentose m/z 413-60-42	[C11H20O10-H]-	311,09837	311,09836	-0,03214
**309,11909**	[OMethyl- 6-deoxyhexose-Hexose -CH2O- H]-	C13H24O10-CH2O-H]-	309,11911	309,11909	-0,06470
	[6-deoxyhexose-6-deoxyhexose -H]-	C12H22O9 -H]-	309,11911	309,11909	-0,06470
**307,06707**	[UA-Pentose -H20-H]- ; [m/z 439- pentose-H2O-H]-	[C11H18O11-H2O-H]-	307,06707	307,06707	0,00000
**293,08778**	[Hexose-Pentose -H2O-H]- m/z 425 -Pentose (relié à m/z 443 et m/z 323 -30: m/z 425 (-90-42)	[C11H20O10 -H2O-H]-	293,08781	293,08778	-0,10236
	[Succinyl- OAc-Hexose- H]-	[C11H18O9-H]-	293,08781	293,08778	-0,10236
**291,10855**	[6-deoxyhexose-6-deoxyhexose -H2O-H]- ou OMethyl-6-deoxyhexose-Hexose-CH2O-H	[C12H22O9-H2O-H]-	291,10854	291,10855	0,03435
	[UA-Deoxypentose-H]- ; m/z 423-Pentose; m/z 407 -deoxypentose	[C11H16O9-H]-	291,07216	291,07217	0,03436
**281,08780**	[Pentose-Pentose-H]- ; m/z 443-Hexose : m/z 413-pentose: m/z 323-42 (OAc)	[C10H18O9-H]-	281,08781	281,08780	-0,03558
**275,07721**	[Succinyl-Hexose-OAc -H2O-H]	[C11H18O9-H2O-H]-	275,07724	275,07721	-0,10906
**263,07723**	[Pentose-Pentose -H20-H]- ; [m/z 425-Hexose-H]- ; m/z395- pentose : m/z 323-60	[C10H18O9-H2O-H]-	263,07724	263,07723	-0,03801
**175,02486**	[UA -H2O-H]- ; m/z 439 - Pentose-Pentose; [m/z 337-Hexose-H2O-H]- ; [m/z 423 -Pentose-deoxypentose-H]-	[C6H10O7-H2O-H]	175,02481	175,02486	0,28567
**161,04560**	[Hexose -H2O-H]- ; [m/z 425-Pentose-Pentose -H]-; m/z 323 -162	[C6H12O6-H2O-H]-	161,04555	161,04560	0,31047

Table S6. Summary of potential structures and number of fragments indicative of these structures

Occurence de fragments	Motif
9	Pentose-Pentose-Hexose
4	Hexose-DiOAc-Hexose
4	Tri-pentose
4	OMe-6-Deoxyhexose-Hexose
3	UA-Pentose-Pentose
3	UA-Hexose-Hexose
3	Succinyl-Hexose-Hexose-OMe
3	UA-6Deoxyhexose-6-Deoxyhexose
3	Hexose-Hexose-Hexose
2	Succinyl-Hexose-OAc

Table S7. ^{1}H and ^{13}C NMR chemical shifts $\delta_{H/C}$ of major ^{1}H, ^{13}C HSQC cross peaks of *R. tataouinensis* EPS (see attendant tables); asterisk denotes superimposed cross peaks at given position of $\delta_{H/C}$.

Figure	number	δ_H [ppm]	δ_C [ppm]	CH_n
Fig. S7	a_1	1.207	19.42	C-CH_3
Fig. S7	a_2	1.120	19.43	C-CH_3
Fig. S7	a_3	1.193	19.37	C-CH_3
Fig. S7	b_1	1.236	22.70	C-CH_3
Fig. S7	b_2	1.228	22.64	C-CH_3
Fig. S7	b_1	1.244	22.81	C-CH_3
Fig. S7	c_1	1.388	17.13	C-CH_3
Fig. S7	c_2	1.340	16.62	C-CH_3
Fig. S7	c_3	1.321	17.46	C-CH_3
Fig. S7	d_1	1.461	22.75	C-CH_2
Fig. S7	d_2	1.428	22.61	C-CH_2
Fig. S7	d_3	1.416	22.58	C-CH_2
Fig. S7	d_4	1.402	22.75	C-CH_2
Fig. S7	d_5	1.387	22.61	C-CH_2
Fig. S7	e_1	1.677	26.89	C-CH_2
Fig. S7	e_2	1.707	26.72	C-CH_2
Fig. S7	e_3	1.656	26.59	C-CH_2
Fig. S7	f_1	1.760	30.99	C-CH_2
Fig. S7	f_2	1.736	30.99	C-CH_2
Fig. S7	f_3	1.747	30.95	C-CH_2
Fig. S7	f_4	1.770	30.82	C-CH_2
Fig. S7	g_1	1.842	30.99	C-CH_2
Fig. S7	g_2	1.815	30.99	C-CH_2
Fig. S7	g_3	1.805	31.02	C-CH_2
Fig. S7	g_4	1.793	31.02	C-CH_2
Fig. S7	g_5	1.856	31.02	C-CH_2
Fig. S7	g_6	1.866	30.78	C-CH_2
Fig. S7	g_7	1.873	30.82	C-CH_2
Fig. S7	h_1	1.938	28.31	C-CH_2
Fig. S7	h_2	1.925	28.31	C-CH_2
Fig. S7	h_3	1.909	27.43	C-CH_2
Fig. S7	h_4	1.944	28.21	C-CH_2
Fig. S7	h_5	1.952	28.52	C-CH_2
Fig. S7	i_1	2.029	28.48	C-CH_2
Fig. S7	i_2	2.013	28.48	C-CH_2
Fig. S7	i_3	2.044	28.01	C-CH_2
Fig. S7	i_4	2.057	27.68	C-CH_2
Fig. S7	j_1	2.037	22.97	C-CH_3
Fig. S7	j_2	2.024	22.83	C-CH_3
Fig. S7	k	2.091	22.96	C-CH_3
Fig. S7	l	2.090	14.74	C-CH_3
Fig. S7	m_1	2.264	34.30	C-CH_2
Fig. S7	m_2	2.270	34.32	C-CH_2
Fig. S7	m_3	2.275	34.29	C-CH_2
Fig. S7	m_4	2.283	34.28	C-CH_2
Fig. S7	m_5	2.254	34.36	C-CH_2
Fig. S7	m_6	2.245	34.19	C-CH_2
Fig. S7	m_7	2.238	34.14	C-CH_2
Fig. S7	m_8	2.232	34.11	C-CH_2
Fig. S7	m_9	2.220	34.27	C-CH_2
Fig. S7	m_{10}	2.209	34.61	C-CH_2

Fig. S7	n_1	2.406	45.70	C-**CH_2**
Fig. S7	n_2	2.421	45.78	C-**CH_2**
Fig. S7	n_3	2.431	45.85	C-**CH_2**
Fig. S7	n_4	2.438	45.84	C-**CH_2**
Fig. S7	o	2.695	33.22	N-**CH**
Fig. S7	p	2.723	33.78	N-**CH**
Fig. S7	q_1	2.986	39.80	N-**CH_2**
Fig. S7	q_2	3.013	39.69	N-**CH_2**
Fig. S7	r	3.007	49.12	N-**CH_2**
Fig. S7	s_1	3.011	49.26	N-**CH_2**
Fig. S7	s_2	3.001	49.06	N-**CH_2**
Fig. S7	s_3	3.005	49.13	N-**CH_2**
Fig. S7	s_4	2.994	48.72	N-**CH_2**
Fig. S8	a	3.481	69.64	O-**CH**
Fig. S8	b	3.499	76.82	O-**CH**
Fig. S8	c	3.481	69.64	O-**CH**
Fig. S8	d	3.628	75.77	O-**CH**
Fig. S8	e	3.781	75.84	O-**CH**
Fig. S8	f	3.812	76.25	O-**CH**
Fig. S8	g	3.78-3.93	70-73	**O-**CH**
Fig. S8	g_1	3.799	71.52	O-**CH**
Fig. S8	h	3.648	63.55	O-**CH_2**
Fig. S8	i	3.758	61.36	**O-**CH_2**
Fig. S8	j	3.897	61.57	**O-**CH_2**
Fig. S8	k	4.018	43.18	N-**CH_2**
Fig. S8	l	4.303	50.29	-CONH-**CaH-**
Fig. S8	m	4.283	54.18	-CONH-**CaH-**
Fig. S8	n_1	4.532	56.25	-CONH-**CaH-**
Fig. S8	n_2	4.495	56.40	O-**CH**
Fig. S8	o	4.224	71.35	O-**CH**
Fig. S8	p	4.064	70.86	O-**CH**
Fig. S8	q	4.114	74.95	O-**CH**
Fig. S8	r	4.115	81.89	O-**CH**
Fig. S8	s	4.024	83.44	O-**CH**
Fig. S9	a_1	4.506	103.33	O_2CH
Fig. S9	a_2	4.514	103.35	O_2CH
Fig. S9	a_3	4.524	102.95	O_2CH
Fig. S9	b_1	4.555	100.74	O_2CH
Fig. S9	b_2	4.533	102.90	O_2CH
Fig. S9	c	4.555	100.73	O_2CH
Fig. S9	d	4.621	100.59	O_2CH
Fig. S9	e_1	4.691	103.86	O_2CH
Fig. S9	e_2	4.685	103.67	O_2CH
Fig. S9	e_3	4.677	103.65	O_2CH
Fig. S9	f_1	4.786	101.11	O_2CH
Fig. S9	f_2	4.797	101.29	O_2CH
Fig. S9	f_3	4.771	101.19	O_2CH
Fig. S9	g_1	4.891	102.42	O_2CH
Fig. S9	g_2	4.887	102.41	O_2CH
Fig. S9	g_3	4.874	102.39	O_2CH
Fig. S9	h_1	5.048	102.26	O_2CH
Fig. S9	h_2	5.040	102.31	O_2CH
Fig. S9	h_3	5.031	101.89	O_2CH
Fig. S9	i_1	5.088	96.56	O_2CH
Fig. S9	i_2	5.080	95.76	O_2CH

Fig. S9	i_3	5.086	95.39	O_2CH
Fig. S9	j_1	5.164	95.70	O_2CH
Fig. S9	j_2	5.169	95.93	O_2CH
Fig. S9	k_1	5.278	97.12	O_2CH
Fig. S9	l_1	5.084	107.83	O_2CH
Fig. S9	l_2	5.048	107.63	O_2CH
Fig. S9	l_3	5.031	108.22	O_2CH
